# Herbal medicine for idiopathic central precocious puberty: A protocol for a systematic review of controlled trials

**DOI:** 10.1097/MD.0000000000010267

**Published:** 2018-03-30

**Authors:** Hye Lim Lee, Yoo Been Lee, Jun-Yong Choi, Ju Ah Lee

**Affiliations:** a Department of Pediatrics, College of Korean Medicine, Gachon University, Seongnam; b School of Korean Medicine, Pusan National University, Yangsan; c Department of Internal Medicine College of Korean Medicine, Gachon University, Seongnam, Korea.

**Keywords:** herbal medicine, idiopathic central precocious puberty, protocol, systematic review

## Abstract

**Background:**

Herbal medicine is widely used in East Asia to treat idiopathic central precocious puberty (ICPP). Most of the available clinical trials that investigated herbal medicine for ICPP have been included in this review. This systematic review will assess the efficacy and safety of herbal medicine for ICPP.

**Methods:**

Eleven databases, including Asian databases, will be searched for studies conducted through 2018. We will include randomized controlled trials assessing herbal medicine for ICPP. The risk of bias will be evaluated using the Cochrane risk of bias assessment tool, and confidence in the cumulative evidence will be evaluated using the Grading of Recommendations Assessment, Development, and Evaluation instrument.

**Ethics and dissemination:**

This systematic review will be published in a peer-reviewed journal and disseminated both electronically and in print. The review will be updated to inform and guide health care practices.

**Trial registration number:**

PROSPER 2018 CRD42018087988.

## Abstract 2

Supplemental Digital Content is available in the text

## 1 Introduction

Central precocious puberty (CPP, also known as gonadotropin-releasing hormone [GnRH]-dependent precocious puberty) is defined as premature activation of the hypothalamic-pituitary-gonadal axis and the development of secondary sexual characteristics in girls before 8 years of age and in boys before 9 years of age.^[1]^ The CPP in the absence of an identifiable cause is termed idiopathic CPP (ICPP); with improvements in the standards of living, the incidence of ICPP has increased in recent years. The incidence of CPP among Korean girls has increased significantly from 3.3 per 100,000 girls in 2004 to 50.4 per 100,000 girls in 2010.^[2]^ Furthermore, the incidence of CPP is much higher in girls, who account for nearly 90% of all ICPP cases.^[3]^ The ICPP is predominantly found in girls, and while the majority of female CPP cases are idiopathic, cases in boys are more frequently secondary to an organic cause.^[4]^

The premature secretion of estradiol increases the growth velocity and accelerates bone age, which can shorten the growing period and result in short adult height. The CPP can cause early menarche in girls and psychosocial problems, especially in those with early onset and rapid progression.^[5]^ Development progresses according to the normal process of sexual development, and the cause of CPP cannot always be determined in the clinic.^[6]^

The GnRH analogue therapy is currently the most common clinical treatment for CPP; such analogues inhibit the secretion of gonadotropin and the production of sex steroids, thereby halting the progression of puberty.^[7]^

Recently, the use of complementary and alternative medicine (CAM) to treat ICPP has increased in East Asia.^[8,9]^ Herbal medicines have been used to delay pubertal development and promote growth in patients with ICPP.^[10]^

Therefore, this study will seek to systematically review randomized controlled trials (RCTs) to assess the efficacy and safety of herbal medicine for the treatment of ICPP. This protocol will describe the methods of searching Asian databases and will therefore permit the inclusion of CAM-related literature that cannot be retrieved from English-language databases.

## 2 Methods

### 2.1 Study registration

This study will follow the guidelines outlined in the Preferred Reporting Items for Systematic Reviews and Meta-Analysis (PRISMA) statement for meta analyses of health care interventions;^[11]^ additionally, the current protocol report adheres to the PRISMA Protocols (PRISMA-P).^[12]^ The protocol for this systematic review has been registered in PROSPERO 2018 under number CRD42018087988.

### 2.2 Ethical approval

Because this study is not a clinical study, ethical approval is not required.

### 2.3 Data sources

The following databases will be searched from inception to the present date: Medline, Embase, the Cochrane Central Register of Controlled Trials (CENTRAL), Alternative and Complementary Medicine DATABASE, and Cumulative Index to Nursing and Allied Health Literature. We will also search 6 Korean medical databases (Oriental medicine Advanced Searching Integrated System, the Korean Traditional Knowledge Portal, the Korean Studies Information Service System, KoreaMed, the Korean Medical Database, and DBPIA); and 3 Chinese databases: CNKI (including the China Academic Journal, the China Doctoral Dissertations and Masters’ Theses Full-text Database, the China Proceedings of Conference Full-Text Database and the Century Journal Project), Wanfang and VIP. In addition, we will search a Japanese database and conduct non-electronic searches of conference proceedings, our own article files, and 9 traditional Korean medicine journals. The Medline database search strategy is presented in Supplement 1. Similar search strategies will be used for the other databases.

## 3 Types of studies

Prospective RCTs and quasi-RCTs that evaluated the efficacy of herbal medicine for ICPP will be included in this review. Both treatment with herbal medicine alone and concurrent treatment with herbal medicine and another therapy will be considered acceptable if herbal medicine is administered to the intervention group only and any other treatment is administered equally to both groups. Trials with any type of control intervention will be included. There will be no restrictions on publication language. Hard copies of all articles will be obtained and read in full.

## 4 Types of participants

This study will include patients who were diagnosed with ICPP. We will exclude patients with CPP secondary to congenital hypothyroidism, germ cell tumors, various types of gonadal tumors, organic diseases including congenital adrenal hyperplasia, adrenal tumors, various intracranial tumors, or central nervous system diseases. Due to the outcome determinations, only girls will be included in the study.

## 5 Types of interventions

Interventions of any formulation (i.e., decoction, tablets, capsules, pills, powders, and extracts) of herbal medicine will be eligible for inclusion. This study will include only previous studies using herbal medicines prescribed by traditional East Asian medicine doctors, and the compositions of the interventions will be reviewed.

## 6 Data extraction and quality assessment

Hard copies of all articles will be obtained and read in full. Two authors (JAL and SJ) will extract the data and assess the quality using a predefined data extraction form. In addition, all interventions that involve acupuncture will be extracted using the Standards for Reporting Interventions in Clinical Trials of Acupuncture. The risk of bias will be evaluated using the Cochrane risk of bias assessment tool, version 5.1.0, which considers random sequence generation, allocation concealment, blinding of participants and personnel, blinding of outcome assessment, completeness of outcome data, selective reporting, and other sources of bias.^[13]^ The results of such evaluations will be presented by utilizing scores of ‘L’, ‘U’, and ‘H’ to indicate a low risk of bias, an uncertain risk of bias, and a high risk of bias, respectively. Disagreements will be resolved by discussion among all authors. When disagreements regarding selection cannot be resolved through discussion, an arbiter (DHL) will make the final decision.

## 7 Data collection and synthesis

### 7.1 Outcome measures

#### 7.1.1 Primary outcomes

The primary outcome will be changes in hormone levels (follicle-stimulating hormone, luteinizing hormone, and estradiol).

#### 7.1.2 Secondary outcomes

The secondary outcomes will include safety, which will be evaluated based on adverse events, and changes in ovarian volume and intrauterine volume.

### 7.2 Assessment of bias in the included studies

We will independently assess bias in the included studies in accordance with the criteria in the Cochrane Handbook, version 5.1.0; these criteria include random sequence generation, allocation concealment, blinding of participants and personnel, blinding of outcome assessment, completeness of outcome data, selective reporting, and other sources of bias.^[13]^

### 7.3 Data synthesis

Differences between the intervention and control groups will be assessed. The mean difference (MD) and 95% confidence interval (CI) will be used to measure the effects of treatment for continuous data. We will convert other forms of data into the MD. For outcome variables on different scales, we will use the standard MD and 95% CI. For dichotomous data, we will present treatment effects as the risk ratio (RR) and 95% CI; other binary data will be converted into the RR.

All statistical analyses will be conducted using the Cochrane Collaboration's software program Review Manager (RevMan) version 5.3 for Windows (Copenhagen, The Nordic Cochrane Centre, the Cochrane Collaboration, 2012). We will contact the corresponding authors of studies with missing information to acquire and verify data whenever possible. As appropriate, we will pool data across studies to conduct a meta analysis using fixed or random effects models. We will use GRADEpro software from Cochrane Systematic Reviews to create a summary table.

### 7.4 Unit of analysis issues

For crossover trials, data from the first treatment period will be used. For trials that assessed more than one control group, the primary analysis will combine data from each control group. Subgroup analyses of the control groups will be performed. Each patient will be counted only once in these analyses.

### 7.5 Addressing missing data

Intention-to-treat analyses that include all randomized patients will be performed. For patients with missing outcome data, last observation carried forward analysis will be conducted. When individual patient data are initially unavailable, we will review the original source and/or published trial reports to obtain these data.

### 7.6 Assessment of heterogeneity

Based on our data analyses, we will use random or fixed effects models to conduct the meta analysis. Chi-squared and *I*^2^ tests will be used to evaluate the heterogeneity of the included studies, with *I*^2^ > 50% indicative of high heterogeneity. When heterogeneity is observed, subgroup analyses will be conducted to explore the possible causes of this heterogeneity.^[14]^

### 7.7 Assessment of reporting biases

Funnel plots will be generated to detect reporting biases when a sufficient number of included studies (at least 10 trials) are available.^[15]^ However, because funnel plot asymmetry is not equivalent to publication bias, we will aim to identify the possible reasons for any asymmetry in the included studies, including small-study effects, poor methodological quality, and true heterogeneity.^[15,16]^

## 8 Discussion

In Asian countries, herbal medicine is a form of complementary medicine that has been widely applied to treat ICPP.^[17,18]^ In recent studies, herbal medicines have been studied as both formulas and as single agents. All formulations of herbal medicine, such as decoctions, tablets, capsules, pills, powders, and extracts, will be considered for inclusion in this review. We will include only studies using herbal medicines prescribed by traditional East Asian medicine doctors and exclude studies on a single herb.

Because of the increased incidence of ICPP and treatment limitations, there is a critical and urgent need to identify and develop safe, complementary and alternative treatments for children with ICPP. Thus far, there have been no systematic reviews of herbal medicine for ICPP. This review will be useful to patients and health care providers in the field of pediatric medicine.

## Author contributions

**Conceptualization:** H.L. Lee.

**Funding acquisition:** J-Y. Choi.

**Project administration:** J.A. Lee.

**Writing – original draft:** H.L. Lee, J.A. Lee.

**Writing – review & editing:** J.A. Lee, J-Y. Choi, Y.B. Lee.

## Supplementary Material

Supplemental Digital Content
