# Supplementary material for: Herbal medicine for idiopathic central precocious puberty: A protocol for a systematic review of controlled trials
Source: Medicine (Baltimore). 2018 Mar 30;97(13):e0267. doi: 10.1097/MD.0000000000010267 (PMC5895428; doi:10.1097/MD.0000000000010267)
Supplement: Supplemental Digital Content [file medi-97-e0267-s001.docx]

Supplement 1. Search strategy

#1 Search "idiopathic central precocious puberty"[Title/Abstract]

#2 Search "precocious puberty"

#3 Search "Puberty, Precocious"[Mesh]

#4 Search (#1 OR #2 OR #3)

#5 Search "Chinese medicine"[Mesh]

#6 Search "Kampo medicine"

#7 Search "Korean medicine"OR "Traditional Korean medicine"

#8 Search ((Plant Extracts"[Title/Abstract]) OR "Herbal Medicine"[Title/Abstract] or "herbal*")) OR (("Plant Extracts"[Mesh]) OR "Herbal Medicine"[Mesh]

#9 #5 OR #6 OR #7 OR #8

#10 #4 AND #9
